# Supplementary figures and images for: Inferring binding specificities of human transcription factors with the wisdom of crowds
Source: bioRxiv. 2025 Nov 17:2025.11.16.688692. Preprint. [Version 1] doi: 10.1101/2025.11.16.688692 (PMC12667932; doi:10.1101/2025.11.16.688692)

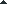

Supplement: Supplement 1 [file media-1.gz › ibis_report/tablesorter-master/dist/css/images/black-asc.gif]

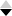

Supplement: Supplement 1 [file media-1.gz › ibis_report/tablesorter-master/dist/css/images/metro-black-desc.png]

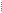

Supplement: Supplement 1 [file media-1.gz › ibis_report/tablesorter-master/dist/css/images/dragtable-handle.png]

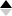

Supplement: Supplement 1 [file media-1.gz › ibis_report/tablesorter-master/dist/css/images/metro-black-asc.png]

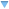

Supplement: Supplement 1 [file media-1.gz › ibis_report/tablesorter-master/dist/css/images/dropbox-desc.png]

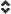

Supplement: Supplement 1 [file media-1.gz › ibis_report/tablesorter-master/dist/css/images/bootstrap-black-unsorted.png]

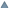

Supplement: Supplement 1 [file media-1.gz › ibis_report/tablesorter-master/dist/css/images/dropbox-asc-hovered.png]

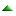

Supplement: Supplement 1 [file media-1.gz › ibis_report/tablesorter-master/dist/css/images/green-asc.gif]

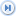

Supplement: Supplement 1 [file media-1.gz › ibis_report/tablesorter-master/dist/css/images/last.png]

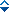

Supplement: Supplement 1 [file media-1.gz › ibis_report/tablesorter-master/dist/css/images/ice-desc.gif]

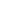

Supplement: Supplement 1 [file media-1.gz › ibis_report/tablesorter-master/dist/css/images/white-desc.gif]

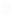

Supplement: Supplement 1 [file media-1.gz › ibis_report/tablesorter-master/dist/css/images/metro-unsorted.png]

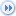

Supplement: Supplement 1 [file media-1.gz › ibis_report/tablesorter-master/dist/css/images/next.png]

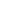

Supplement: Supplement 1 [file media-1.gz › ibis_report/tablesorter-master/dist/css/images/bootstrap-white-unsorted.png]

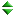

Supplement: Supplement 1 [file media-1.gz › ibis_report/tablesorter-master/dist/css/images/green-unsorted.gif]

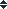

Supplement: Supplement 1 [file media-1.gz › ibis_report/tablesorter-master/dist/css/images/black-unsorted.gif]

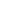

Supplement: Supplement 1 [file media-1.gz › ibis_report/tablesorter-master/dist/css/images/white-asc.gif]

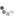

Supplement: Supplement 1 [file media-1.gz › ibis_report/tablesorter-master/dist/css/images/metro-loading.gif]

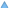

Supplement: Supplement 1 [file media-1.gz › ibis_report/tablesorter-master/dist/css/images/dropbox-asc.png]

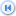

Supplement: Supplement 1 [file media-1.gz › ibis_report/tablesorter-master/dist/css/images/first.png]

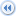

Supplement: Supplement 1 [file media-1.gz › ibis_report/tablesorter-master/dist/css/images/prev.png]

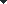

Supplement: Supplement 1 [file media-1.gz › ibis_report/tablesorter-master/dist/css/images/black-desc.gif]

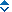

Supplement: Supplement 1 [file media-1.gz › ibis_report/tablesorter-master/dist/css/images/ice-asc.gif]

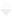

Supplement: Supplement 1 [file media-1.gz › ibis_report/tablesorter-master/dist/css/images/metro-white-desc.png]

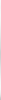

Supplement: Supplement 1 [file media-1.gz › ibis_report/tablesorter-master/dist/css/images/green-header.gif]

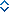

Supplement: Supplement 1 [file media-1.gz › ibis_report/tablesorter-master/dist/css/images/ice-unsorted.gif]

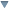

Supplement: Supplement 1 [file media-1.gz › ibis_report/tablesorter-master/dist/css/images/dropbox-desc-hovered.png]

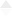

Supplement: Supplement 1 [file media-1.gz › ibis_report/tablesorter-master/dist/css/images/metro-white-asc.png]

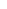

Supplement: Supplement 1 [file media-1.gz › ibis_report/tablesorter-master/dist/css/images/white-unsorted.gif]

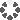

Supplement: Supplement 1 [file media-1.gz › ibis_report/tablesorter-master/dist/css/images/loading.gif]

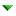

Supplement: Supplement 1 [file media-1.gz › ibis_report/tablesorter-master/dist/css/images/green-desc.gif]
